# Inhalation of Essential Oil from Mentha piperita Ameliorates PM10-Exposed Asthma by Targeting IL-6/JAK2/STAT3 Pathway Based on a Network Pharmacological Analysis

**DOI:** 10.3390/ph14010002

**Published:** 2020-12-22

**Authors:** Mi Hye Kim, Sang Jun Park, Woong Mo Yang

**Affiliations:** Department of Convergence Korean Medical Science, College of Korean Medicine, Kyung Hee University, 26 Kyungheedae-ro, Dongdaemun-gu, Seoul 02447, Korea; kimmihye526@khu.ac.kr (M.H.K.); everythingisok@khu.ac.kr (S.J.P.)

**Keywords:** *Mentha* essential oil, peppermint oil, asthma, particulate matter

## Abstract

Fine particulate matter (PM) exposure exhibits a crucial risk factor to exacerbate airway epithelial remodeling, fibrosis, and pulmonary destruction in asthma. Based on the use of essential oils from aromatic plants on pain relief and anti-inflammatory properties, we investigated the inhibitory effects of essential oil derived from the Mentha species (MEO) against asthma exposed to PM10. The MEO (0.1 *v*/*v* %) was aerosolized by a nebulizer to ovalbumin and PM10-induced asthmatic mice. Histological changes were confirmed in the lung tissues. To define the mode of action of the MEO on asthma, a protein–protein interaction network was constructed using menthol and menthone as the major components of the MEO. Cytokine expression and the JAK2/STAT3 signaling pathway were analyzed in lung epithelial A549 cells co-treated with MEO and PM10. Inhalation of MEO by nebulization inhibited respiratory epithelium hyperplasia, collagen deposition, and goblet cell activation in asthmatic mice. Through a network pharmacological analysis, cytokine–cytokine receptor interaction and JAK/STAT was expected to be underlying mechanisms of MEO on asthma. Treatment with MEO significantly reduced the IL-6 levels with a decrease in pro-inflammatory and T helper 2-specific cytokines. PM10-induced phosphorylation of JAK2 and STAT3 was significantly decreased by MEO. Collectively, MEO may have an inhibitory effect on asthma under the condition of PM10 exposure through the IL-6/JAK2/STAT3 signaling pathway.

## 1. Introduction

Asthma is one of the major allergic respiratory diseases with various symptoms including wheezing, breathlessness, chest tightness and/or coughing, which are induced by airway hyperresponsiveness and pulmonary inflammation, and remodeling [1]. Fine particulate matter (PM) consisting of solid and liquid particles such as black carbon, metals, nitrate, sulfate, polycyclic aromatic hydrocarbons, and automobile exhaust particles have recently been reported to exacerbate respiratory diseases such as asthma, rhinitis, bronchitis, chronic obstructive pulmonary disease (COPD), and hospital admissions as an allergen and, furthermore, are carcinogenic to humans, especially in the lungs [2]. Pollutants such as respirable particulate matter PM_10_, fine particulate matter PM_2.5_, NO_2_, SO_2_, and O_3_, can possibly invade the airways entering the alveolar tissue, which is an important risk factor in the management of asthmatic patients as it lowers their quality of life [3].

Diverse potential molecular mechanisms have been suggested to clarify the relationship of air pollution exposure with allergic sensitization and asthma exacerbation such as oxidative stress, DNA damage, micronucleus formation, and stimulation of pro-inflammatory factors. Asthma is generally thought to be T helper (Th)-2 pathway-mediated inflammatory disease, which is also triggered by environmental PM [4]. Glencross et al. have suggested that pro-inflammatory cytokines, especially IL-6, are clinically relevant to particulate in the rodent model and humans [5]. A number of studies showed IL-6 leads to the activation of the Janus kinase (JAK) family, and then mediates the phosphorylation of the major transcriptional factor signal transducer and transcription 3 (STAT3) [6]. The STAT3 transcription factor is proposed to be one of the initial activators of Th2 cell program in allergic lung inflammation [7]. Additional research has reported that atmospheric diesel exhaust particulate exposure induces the activation of STAT3 in primary cultures of human airway epithelial cells [8]. Moreover, there is an investigation to find the effects of PM_2.5_ on JAK2/STAT3 signaling pathway in human bronchial epithelial cells [9], giving the possibility of a therapeutic target for asthma by inhibiting the JAK/STAT signaling pathway under an air pollution-exposed condition.

Inhalation of essential oils from aromatic plants by an aerosol spray, nebulizer, or nasal treatment has been regarded as a health enhancer due to their pain relief, and anti-inflammatory and anti-tumor properties [10]. Genus *Mentha* is known as an aromatic species used for food flavoring and medicinal herbs worldwide, because the leaves, flowers, and stems of this genus have been known to contain terpenes/essential oils [11,12]. Essential oils (EOs) of the *Mentha* species, regarded as peppermint oil, was reported to ameliorate histamine and ovalbumin (OVA)-induced bronchial asthma [13]. Interestingly, healthy adults nebulized with *Mentha* EOs (MEO) for 5 min following 1500 m running tests showed significant improvement in lung function and exercise performance [14]. There is accumulating evidence regarding the use of essential oils against urban air pollution to reduce the risk of respiratory diseases [15].

In the present study, we conducted a protein–protein interaction network (PPIN) analysis, an effective pharmacological approach to predict the mechanism of a drug target. Module (M)-network and gene ontology (GO) analysis could offer the molecular mechanism of the anti-asthmatic action of MEO, which is associated with the JAK/STAT pathway. Then, we verified the inhibitory effects of MEO on allergic asthma under the condition of PM_10_ exposure by verifying the inhibition of the JAK2/STAT3 signaling pathway and its series Th2-specific cytokine production in vivo and in vitro levels.

## 2. Results

### 2.1. Construction of Menthol and Menthone Network

**Figure 1:**
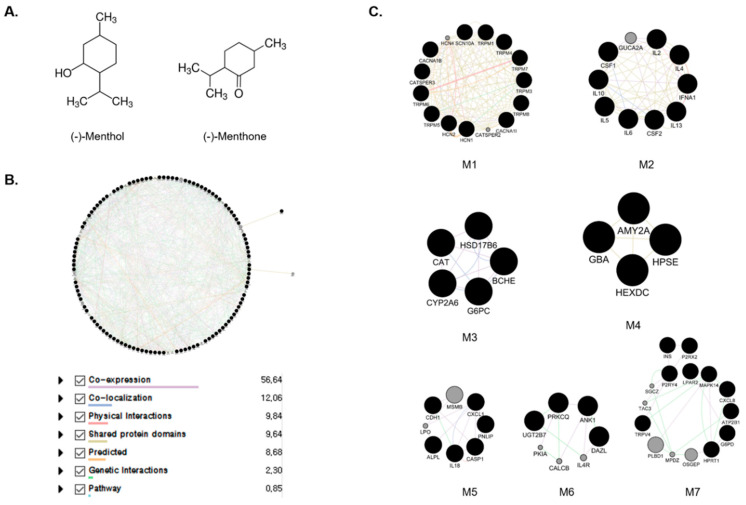
The structures of menthol and menthone, major components of *Mentha* essential oil (**A**). Protein–protein interaction network of *Mentha* essential oils (MEO) (**B**). The nodes and edges indicate the proteins and their relationships. Module extracted by Gene ontology enrichment analysis (**C**). The black nodes present seed nodes and the gray ones are nodes that interact with the seed nodes. Purple, Co-expression; Blue, Co-localization; Pink, Physical interactions; Yellow, shared protein domains; Orange, Predicted; Green, Genetic interactions; Sky, pathway.

There are 100 co-efficient genes and 86 co-efficient genes for menthol and menthone, respectively through PubChem system. In total, 186 genes were yielded to be the targets of MEO. In the process of Protein–Protein Interactions (PPI) construction through Genemania, 186 targets were sorted to 108 targets with the score ≥0.7, and 20 targets were added, which mainly related to the 108 targets. Total 128 targets were used to create the network of menthol and menthone, which are two main components of MEO (Table S1). With the 128 targets of MEO, we performed a protein interaction network, showing interconnectivity either by reported co-expression, co-localization, physical interactions, shared protein domains, predicted, genetic interactions and pathways. The edges showed different correlations between nodes. Through complex interactions involving co-expression, co-localization, physical interactions, shared protein domains, predicted, genetic interactions and pathways, protein interaction network established a total 128 nodes and 1103 edges (Figure 1B). Based on this network, functional modules of menthol and menthone were explored by MCODE. 128 targets of MEO were divided into 7 modules including M1, Mineral absorption; M2, Cytokine–cytokine receptor interaction; M3, Retinol metabolism; M4, Other glycan degradation; M5, Legionellosis; M6, Th1, and Th2 cell differentiation; M7, Neuroactive ligand–receptor interaction as represented by KEGG pathway 2019 Human (Figure 1C). GO term of each module were also sorted by *p* value. The most significant term within a module was selected as the one representing the module (Table 1). Detailed Node IDs of the biological process according to the functional modules were described in Table S2.

### 2.2. Module Selection and Cluster of the Enriched Pathway Analysis Using Predicted Targets of Menthol and Menthone

**Figure 2:**
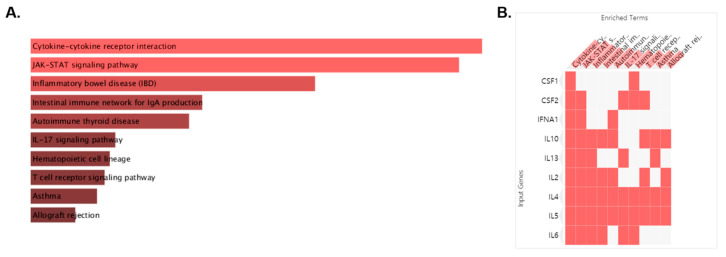
Pathway analysis performed on the “Enrichr” platform. The enriched pathway terms are displayed in a bar graph (**A**). The length of the bar and the brightness of its color represent the significance of the specific pathway. Clustergram of pathways of MEO (**B**). Those clusters are ranked by *p* value. The colors of the boxes represent each cluster.

Among the modules, M2 is cytokine–cytokine receptor interaction, which is closely related to the targets affecting asthma. With 10 targets of M2 including IL2, IL4, IFNA1, IL13, CSF2, IL6, IL5, IL10, CSF1, and GUCA2A, we conducted pathway analysis, which was visualized as bar graph, table and clustergram sorted by *p* value. We found that the first cluster of M2 was cytokine–cytokine receptor interaction and the second that was JAK-STAT signaling pathway (Figure 2A; Table 2). For Cytokine–cytokine receptor interaction, CSF1, CSF2, IFNA1, IL-10, IL-13, IL-2, IL-4, IL-5, and IL-6 were related. For JAK-STAT signaling pathway, targets for cytokine–cytokine receptor interaction except CSF1 were related (Figure 2B). For this, we could find that the target of M2 affecting asthma were cytokine activation and JAK-STAT signaling pathway among the targets of MEO.

### 2.3. Effects of Aerosolized MEO on Histological Changes in PM_10_-Exposed Lung Tissues of Mice

**Figure 3:**
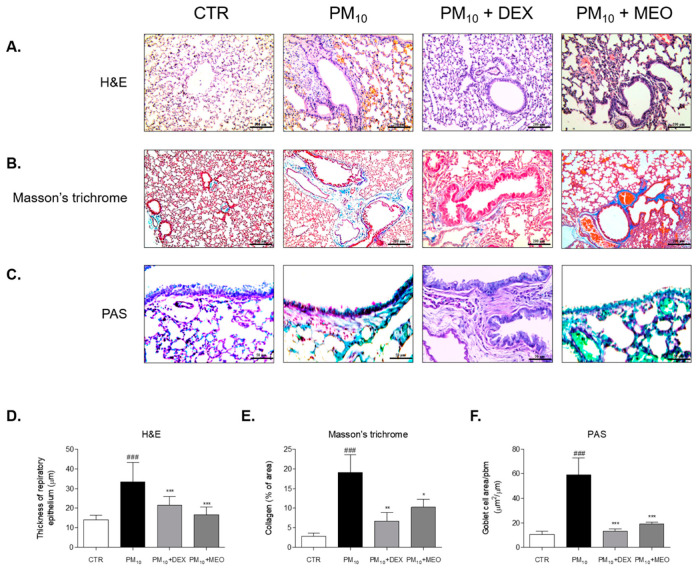
Histological changes of lung tissues stained by Hematoxylin and Eosin (**A**), Masson’s trichrome (**B**) and Periodic acid–Schiff (PAS) (**C**) in ovalbumin (OVA) and PM_10_-exposed mice. Thickness of respiratory epithelium (**D**), collagen deposition % of area (**E**) and goblet cell number per area (**F**) were shown as relative quantified values. The slides of each mice (*n* = 7) were evaluated by randomly selecting 3 photographs and a total 21 images were quantified using Image J computerized densitometry system. Results are presented as mean ± standard error of the mean. ^###^ *p* < 0.001 vs. CTR group; * *p* < 0.05, ** *p* < 0.01 and *** *p* < 0.001 vs. PM_10_ group.

The thickness of epithelial cells in the lung tissues of the PM_10_ group was 2.3 times higher than that of the control (CTR) group. The administration of dexamethasone (DEX) to the PM_10_-challanged mice significantly reduced the thickness of epithelium of lung. Airway wall thickening by PM10 exposure was attenuated by MEO treatment (Figure 3A). There were 35.3% and 50.3% decreases in epithelial thickness in DEX and MEO-aerosolized mice, respectively, compared to PM_10_-exposed mice (Figure 3D). Collagen deposition was increased in the lungs of the PM_10_ group than in the CTR group. Nebulizing with DEX and MEO exhibited reduction of blue-stained collagen area in lung tissues (Figure 3B). While DEX treatment showed 64.7% decrease of collagen deposition, treatment of MEO by nebulizing in PM_10_-exposed mice significantly attenuated that by 46.3% (Figure 3E). In addition, the number of goblet cells was 5 times elevated in the lungs of the PM_10_ group than in the CTR group, which was reduced by administration of DEX. We observed that increased goblet cells were markedly suppressed by MEO aerosolization (Figure 3C). The reduction rate of MEO on goblet cells area/ppm was 67.8% (Figure 3F).

### 2.4. Effects of MEO on Inflammatory Cytokines Levels in PM_10_-Sensitized A549 Cells

**Figure 4:**
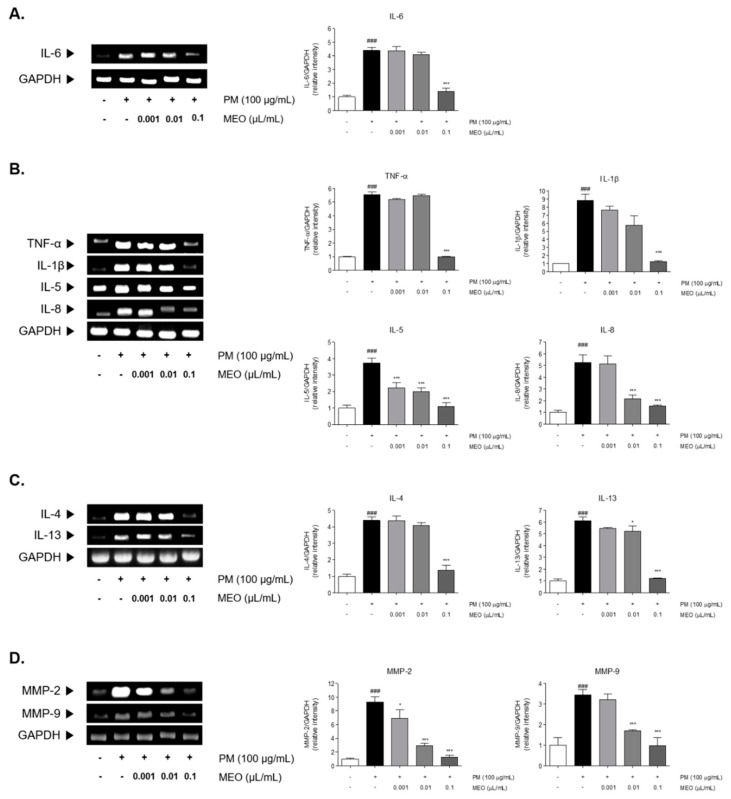
The mRNA expressions of IL-6 (**A**), inflammatory cytokines including TNF-α, IL-1β, IL-5 and IL-8 (**B**), Th2-specific cytokines including IL-4 and IL-13 (**C**), and MMP-2 and MMP-9 (**D**) in PM_10_-exposed A549 lung epithelial cells. The experiments were carried out in triplicate measurements. Results are presented as mean ± standard error of the mean. ^###^ *p* < 0.001 vs. non-treated cells; * *p* < 0.05, and *** *p* < 0.001 vs. PM_10_-treated cells.

PM_10_ exposure to A549 lung cancer cells induced elevation of inflammatory cytokines mRNA levels. Sensitization of PM_10_ significantly elevated the 4.4-fold level of IL-6 in comparison with the non-treated cells. Increased IL-6 level by PM_10_ was significantly decreased by 68.5% in 10^−3^ /mL (*v*/*v* %) of MEO-treated cells (Figure 4A). After PM_10_ exposure for 24 h, mRNA expression of the pro-inflammatory cytokines including TNF-α (5.5-fold) and IL-1β (8.8-fold) were increased in A549 lung cancer cells. Treatment of 10^−3^ /mL (*v*/*v* %) of MEO significantly reduced the expressions of TNF-α and IL-1β by 81.9% and 85.8%, respectively, in PM_10_-exposed cells. Similar effects were shown in the results of IL-5 and IL-8 mRNA expressions. PM_10_-induced increase of IL-5 level was reduced by 10^−3^ /mL (*v*/*v* %) of MEO treatment. Moreover, there were about 58.9% and 70.4% decreases of IL-8 expressions in MEO 10^−4^ and 10^−3^ /mL (*v*/*v* %)-treated cells (Figure 4B). In addition, Th2-specific cytokines, IL-4 and IL-13, were elevated 4.4 and 6.1 times by PM_10_ sensitization compared to non-treated cells. The mRNA level of IL-4 in MEO 10^−4^ /mL (*v*/*v* %)-treated cells was lower than only PM_10_-exposed cells. IL-13 expressions were significantly altered by both 10^−4^ and 10^−3^ /mL (*v*/*v* %) of MEO-treated cells (Figure 4C).

### 2.5. Effects of MEO on Proliferative MMPs, Especially MMP-2 and MMP-9, Levels in PM_10_-Sensitized A549 Cells

There were 9.3, 3.5, and 18.2 times increases in MMP-2 and MMP-9 levels in PM_10_-exposed cells. The treatment of 10^−5^, 10^−4^, and 10^−3^ /mL (*v*/*v* %) of MEO significantly decreased the MMP-2 mRNA expressions in PM_10_-treated cells by 26.0%, 68.5%, and 86.6%. Also, the production of MMP-9 in MEO-treated cells at the concentrations of 10^−4^ and 10^−3^ /mL (*v*/*v* %) was lower than in the PM_10_-treated cells (Figure 4D).

### 2.6. Effects of MEO on JAK/STAT3 Signaling Pathway in PM_10_-Sensitized A549 Cells

**Figure 5:**
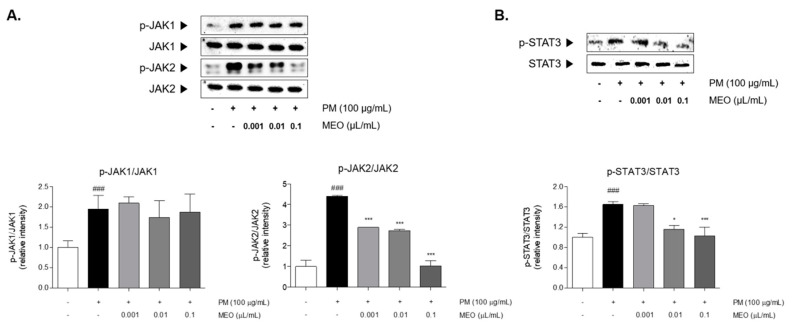
The protein expressions of JAK1 and JAK2 in PM_10_-exposed A549 lung epithelial cells (**A**). The protein expression of STAT3 in PM_10_-exposed A549 lung epithelial cells (**B**). The experiments were carried out in triplicate measurements. Results are presented as mean ± standard error of the mean. ^###^ *p* < 0.001 vs. non-treated cells; * *p* < 0.05, ** *p* < 0.01 and *** *p* < 0.001 vs. PM_10_-treated cells.

The JAK1 and JAK2 were phosphorylated by stimulating PM_10_ in A549 cells. Increase rates of JAK1 and JAK2 by PM_10_ exposure were 2.0 and 4.4 times compared to non-treated cells. The phosphorylation of JAK2 was significantly decreased in the 10^−^^5^, 10^−4^ and 10^−3^ /mL (*v*/*v* %) of MEO-treated cells by 34.0%, 37.7% and 76.4%, respectively, compared to the only PM_10_-treated cells. However, the phosphorylated JAK1 was not altered by MEO treatment at all concentrations compared to PM_10_-treated cells (Figure 5A). Additionally, PM_10_ incubation resulted in STAT3 phosphorylation by 1.4 times compared to non-treated cells. The treatment of MEO in the 10^−4^ and 10^−3^ /mL (*v*/*v* %)-treated cells markedly the phosphorylated levels of STAT3 by 29.9% and 37.7%, respectively (Figure 5B).

### 2.7. Effects of MEO on NF-κB Translocation into Nucleus in PM_10_-Sensitized A549 Cells

**Figure 6:**
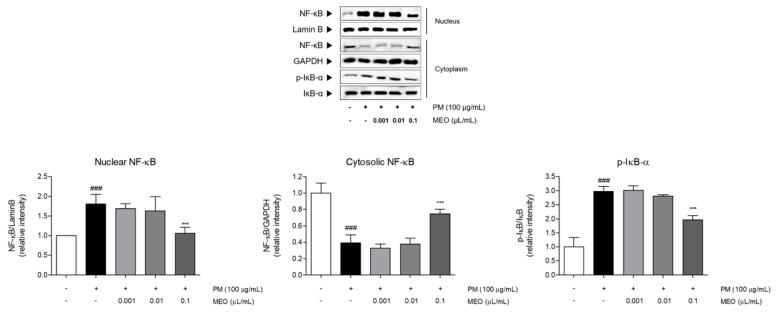
The protein expressions of nuclear NF-κB, cytosolic NF-κB, and IκB-α in PM_10_-exposed A549 lung epithelial cells. The experiments were carried out in triplicate measurements. Results are presented as mean ± standard error of the mean. ^###^ *p* < 0.001 vs. non-treated cells; * *p* < 0.05, ** *p* < 0.01 and *** *p* < 0.001 vs. PM_10_-treated cells.

PM_10_-treated A549 cells exhibited the increase of NF-κB levels in nucleus as well as phosphorylation of IκB-α in cytosol compared to non-treated cells. Increase rates of nucleic NF-κB and phosphorylated IκB-α compared with non-treated cells were 1.8 and 3.0 times. Whereas the expression of NF-κB in cytosol was markedly decreased under exposure of PM_10_ by about 70%. Treatment with 10^−3^ /mL (*v*/*v* %) of MEO significantly down-regulated the NF-κB levels by 41.1% in nucleus, while up-regulated that by 94.4% in cytoplasm. In addition, the phosphorylated IκB-α in cytosol in the MEO-treated cells was 34.4% lower than the only PM_10_-treated cells (Figure 6).

## 3. Discussion

Airway inflammation and remodeling are major features of respiratory diseases in response to air pollution [16]. A number of studies have reported that environmental pollutants negatively cause abnormalities in the respiratory system such as asthma, chronic obstructive pulmonary disease, and lung cancer, because the toxicity of air pollutants inhalation influences the pulmonary epithelium, which has a crucial role in the homeostasis of the lungs [17,18]. Additionally, it is well known that air pollutants exacerbate the damage of pulmonary epithelial cells, leading to airway epithelial remodeling, fibrosis, malignant transformation and pulmonary destruction in asthma [19]. Exposure to PM_10_ induced the airway epithelium of the lung tissues to become thicker than normal epithelial cells. In addition, Masson’s trichrome-stained collagen deposition indicated by blue intensity occurred in the PM_10_-affected fibrosis of the lung. Aerosolization of MEO through the nebulizer significantly decreased the thickness of the pulmonary epithelium, and the accumulation of airway collagen in the OVA and PM_10_-exposed lung tissues. Furthermore, there is a significant correlation between exposure to air pollution and the proportion of goblet cells [20]. Quantification of functional changes in the airway collagen content and goblet cell population as confirmed by airway reactivity to PM challenge showed MEO may contribute to the homeostasis of the lungs, implicating the potential of MEO inhalation as a therapeutic for PM_10_-affected asthma.

To clarify the underlying mechanism of MEO on epithelial remodeling and collagen deposition in asthma, a PPI obtained from menthol and menthone as the main components of MEO was constructed based on a network pharmacological analysis. The pathway analysis of the PPI network of MEO revealed that cytokine–cytokine receptor interactions and the JAK-STAT signaling pathway were expected to be related to the improvement of asthma by MEO inhalation. Asthma is a typical disease associated with the immune responses of Th2 cells. The production of inflammatory cytokines and mediators at the early stage triggers allergic sensitization, recruitment of eosinophils and neutrophils, and augmented Th2-specific cytokines release. Especially, IL-6-mediated JAK/STAT3 signaling has been proven to play crucial roles in the airway remodeling of asthma [21,22]. Release of IL-6 leads to hyperactivation of JAK/STAT3 by phosphorylation [23]. Growing evidence has shown that ambient PM exposure can trigger lung epithelial remodeling by invading the smallest airways [24,25,26]. Based on the result from the network pharmacological analysis using menthol and menthone of peppermint oil, the roles of IL-6 pro-inflammatory cytokines and JAK/STAT3 activation were explored in the present study. In this study, A549 lung carcinoma cells exposed to PM_10_ showed increases of IL-6 and phosphorylation of JAK1/JAK2/STAT3 signaling molecules. MEO treatment of PM_10_-exposed lung epithelial cells significantly inhibited the expressions of IL-6. In addition, the phosphorylated STAT3 levels mediated by PM_10_ were markedly decreased in the MEO-treated cells. STAT3 is phosphorylated by JAK family, including JAK1, JAK2, and tyrosine kinases 2 [27]. To verify upstream kinases that are responsible for phosphorylation of STAT3, we analyzed the both JAK1 and JAK2 protein expressions in PM_10_-treated cells. The present study found that MEO treatment affected the phosphorylation of JAK2, rather than phosphorylation of JAK1. Taken together, those results suggest that MEO could inhibit the IL-6-mediated JAK2/STAT3 signaling pathway in PM_10_-induced airway remodeling.

Th2 cells are known to be major orchestrators in pathogenesis of asthma [28]. The role of STAT3 is well known as an epithelial regulator of the allergic response via recruitment of Th2-specific cytokines [29]. It has been demonstrated that the inflammatory cytokines IL-5 and IL-8 are released in A549 human lung cells after treatment with PM_10_ [30]. Furthermore, Th2-specific IL-4 and IL-13 cytokines were produced by bronchial epithelial cells via STAT3-dependent allergic inflammation in asthma [31]. Those inflammatory cytokines have been importantly related to the development of asthma to up-regulate the STAT3 signals [32]. We observed the inhibition of pro-inflammatory TNF-α, IL-1β, IL-5, and IL-8 cytokines in A549 cells treated with MEO and PM_10_ together compared with PM_10_ only treated cells. Th2 cell-mediated IL-4 and IL-13 expressions were also reduced after the treatment with MEO and PM_10_. Moreover, MMP-2 and MMP-9 activities have been found to participate in the progression of asthma and especially shown to be involved in matrix remodeling and inflammation [33]. Based on the previous reports regarding the role of air pollutants on the MMP activity in pulmonary diseases, we investigated the MMP-2 and MMP-9 expressions in PM_10_-exposed A549 lung cancer cells. The MEO treatment significantly alleviated the PM_10_-induced expressions of MMP-2 and MMP-9 in the A549 cells. Taken together, MEO appears to be effective in regulating the PM-associated airway remodeling by its inhibitory activity on IL-6/JAK2/STAT3-mediated cytokine production.

Several cellular mechanisms, including STAT3, are involved in the pathogenesis of PM-exacerbated respiratory diseases. The NF-κB pathway can enhance tumor development and maintain the production of tumor cells [34]. It has been shown that PM treatment of A549 lung cancer cells resulted in the translocation of NF-κB into the nucleus, while IκB-α is phosphorylated, leading to the inactivation of the NF-κB-IκB-α complex. In addition, the activation of STAT3 mediated by IL-6 can bind to the NF-κB p65 subunit, leading to its activation [35]. In the present study, cells exposed to PM_10_ and MEO showed inactivation of the NF-κB-IκB-α complex. MEO regulated the translocation of NF-κB into the nucleus by reducing the phosphorylation of IκB-α simultaneously.

**Figure 7:**
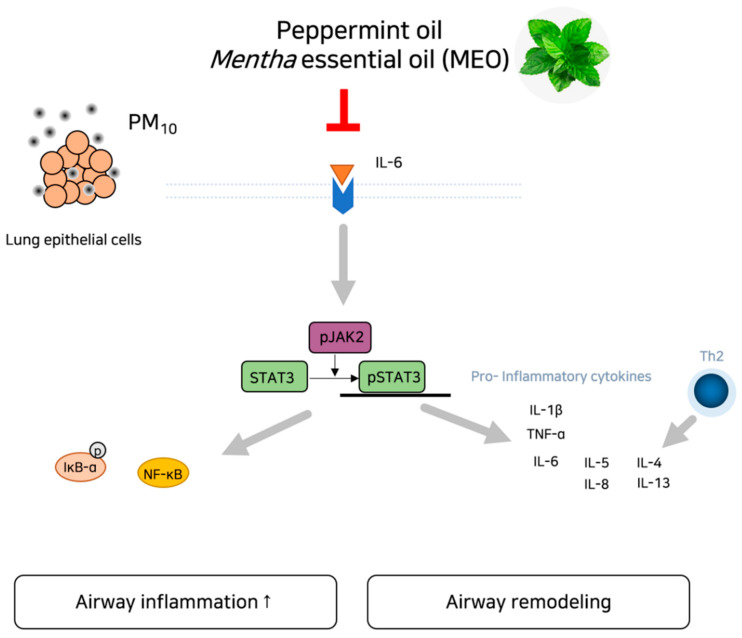
Schematic diagram of potential action of MEO on asthma under exposure of PM via inhibition of IL-6/JAK2/STAT3 pathway.

Given that inflammation and the JAK/STAT3 signaling pathway are potential mechanisms associated with PM_10_, we hypothesized that inhalation of the essential oil derived from the *Mentha* genus, peppermint oil, could inhibit the epithelial remodeling and inflammation in asthma via regulation of IL-6/JAK2/STAT3 signaling pathway according to the network pharmacological analysis. Our results found that PM_10_-induced IL-6 production and JAK2/STAT3 phosphorylation were declined by the MEO treatment, accompanying a decrease in inflammatory responses including cytokines, MMP activation and NF-κB translocation (Figure 7). Consequently, aerosolization of peppermint oil by a nebulizer markedly ameliorated the airway epithelium damage against PM_10_ such as epithelial remodeling, collagen deposition, and goblet cell hyperactivation in asthma. These findings provide a potential property of MEO for the respiratory system in response to air pollution exposure. Peppermint oil would help to alleviate asthma by inhibiting the IL-6/JAK2/STAT3 pathway.

## 4. Materials and Methods

### 4.1. Network Construction

Two main components of MEO, menthol and menthone, were candidate compounds of peppermint oil for network construction [36]. Information and target of the menthol (PubChem ID: 1254) and menthone (PubChem ID: 26447) were extracted from PubChem (https://pubchem.ncbi.nlm.nih.gov/) and summed (Figure 1A). Using GeneMania, we sorted the targets and create PPI. The condition for sorting targets was the score ≥0.7, which means high confidence [37]. Then, PPIN was constructed with the targets sorted by GeneMania using a Cytoscape software according to the previous report [38].

### 4.2. Pathway Analysis and Module Analysis

Based on the protein network constructed above, gene set enrichment analysis (GSEA) based on KEGG pathway 2019 Human (https://www.kegg.jp/) was conducted using Enrichr (https://amp.pharm.mssm.edu/Enrichr/). The pathways were sorted by *p* value, which is the *p* value computed using Fisher’s exact test to assess the deviation from the expected rank. To see the detailed network of MEO, we performed a module division and analysis using a Molecular Complex Detection (MCODE) in Cytoscape. The condition for division was node score cut off = 0.2, K-core = 2, and degree cutoff = 2. For the modules of MEO, after selecting module (M) 2, we conducted pathway analysis based on KEGG pathway 2019 Human, and also GO term analysis based on GO Biological Process 2018 (http://geneontology.org/) in Enrichr. The KEGG pathway and GO term of each module were sorted by *p* value. We excluded *p* value > 0.05 of KEGG pathway and GO term in system and selected one representing term, which had lowest *p* value in each module. Clustergram of pathways and genes was also constructed in Enrichr.

### 4.3. Peppermint Oil Extraction from Mentha Piperita Linn

The leaves of *Mentha piperita* Linn. was purchased from Dong-Yang Herb Inc. (Seoul, Korea). The essential oil extraction was proceeded by hydrodistillation method. Briefly, 100 g of *M. piperita* Linn. was placed within a 1 L round-bottom flask with clevenger apparatus. Steam distillation was performed for 6 h at 100 °C and the obtained volatile oil was 1.6 mL (yield of MEO: 1.60%). A voucher specimen (01-02-01-KR-191217) was deposited at Department of Convergence Korean Medical Science, College of Korean Medicine, Kyung Hee University, Seoul, Korea.

### 4.4. PM_10_-Exposed Animal Treatment

The experimental protocols were approved by the Institutional Animal Ethics Committee of Kyung Hee University in Korea (KHUASP(SE)-19-098; Seoul, Korea). Female BALB/c mice aged 5 weeks were obtained from Raonbio Inc. (Yongin, Korea) and housed in plastic cages. All mice were kept under a 12 h light/dark cycle at a temperature of 22 ± 2 °C and relative humidity of 55 ± 10%. After 1 week of accumulation, mice were randomly subjected into 4 groups (*n* = 7); (1) CTR, normal mice, (2) PM_10_, PM_10_ and ovalbumin (OVA)-exposed mice treated with vehicle, (3) PM_10_ + DEX, PM_10_ and OVA-exposed mice treated with DEX as positive control and (4) PM_10_ + MEO, PM_10_ and OVA-exposed mice treated with MEO. All mice except CTR group were intraperitoneally injected 10 μg OVA emulsified in 500 μg aluminum hydroxide with a total volume of 0.1 mL saline on day 0, 7, and 15. Following the sensitization, mice were challenged with 1 mg OVA and 100 μg PM_10_ supplemented in 50 μL saline by intranasal instillation on day 21, 22. To inhale saline, DEX and MEO, we self-manufactured exposure chamber with nebulizer (Philips, Amsterdam, Netherlands). The tips of 50 mL conical tubes were cut into 1 cm and sealed with plastic round container. The container was connected to nebulizer, and then mice were loaded into the conical tubes to expose the vapor [39]. Sprayed amount of nebulizer was 1 mL/min. CTR and PM_10_ groups were received saline. DEX at a concentration of 2 mg/kg (calculated to 0.06% in saline) was treated to mice to PM_10_ + DEX group by nebulizer. The mice of PM_10_ + MEO group were nebulized with 0.1 *v*/*v* % of MEO in self-manufactured exposure chamber. All treatments were performed for 5 min once a day from day 0 to day 23. At the end of the experiment on day 24, mice were sacrificed.

### 4.5. Histology

Lung tissues were fixed in 10% neutralized formalin for 24 h and then dehydrated with ethanol and xylene. After embedding in paraffin, specimens were cut into 5 μm thickness. Each slide was stained using Hematoxylin and Eosin solution, Masson’s trichrome staining solution and Periodic acid–Schiff (PAS) solution, respectively, according to the manufacture’s instruction. Histopathological changes were examined using the Leica Application Suite (LAS; Leica Microsystems, Buffalo Grove, IL, USA). The thickness of epithelium and blue-stained collagen deposition was determined using an Image J computerized densitometry system. The number of goblet cells was measured in the entire fields of slide for each sample (*n* = 7).

### 4.6. PM_10_-Exposed in Vitro Experiments

The human pulmonary epithelial carcinoma-derived A549 lung epithelial cell line was kindly provided from Prof. Ahn, Kyung Hee University, Seoul, Republic of Korea. The cells were cultured in Dulbecco′s Modified Eagle′s Medium (Gibco; Thermo Fisher Scientific, Inc., Waltham, MA, USA) supplemented with 10% fetal bovine serum and 1% penicillin/streptomycin at 37 °C and 5% CO_2_ atmosphere. A549 cells were seeded in 6 well plates at 7.5 × 10^5^ cells/well until 80% confluence. Then, cells were exposed to 100 μg/mL of PM_10_ in the presence of 10^−5^, 10^−4^ and 10^−3^ /mL (*v*/*v* %) of MEO for 24 h. Urban dust SRM 1649b, as known as PM_10_, was provided from the National Institute of Standards and Technology (Gaithersburg, MD, USA).

### 4.7. Western Blot Analysis

After treatment with PM_10_ and MEO, A549 cells were cultured and lysed with radioimmunoprecipitation assay buffer (50 mM Tris–HCl (pH 7.4), 1% Nonidet P-40, 0.5% sodium deoxycholate, 150 mM NaCl) supplemented with a protease inhibitor (Roche, Hoffmann, Valdese, NC, USA). The quantified 10 μg of protein by Bradford method was separated by sodium dodecyl sulfate–polyacrylamide gel electrophoresis. Then, separated proteins were transferred onto a polyvinylidene difluoride membrane. 3% bovine serum albumin in Tris-Buffered Saline with 0.1% Tween 20 was incubated in membrane to reduce the non-specific antibody binding. Membranes were incubated overnight with primary antibodies (Cell signaling, Beverly, MA, USA) at 4 °C and continued to incubate with horseradish peroxidase-conjugated secondary antibodies (Cell signaling). Signals were detected with an enhanced chemiluminescence detection system (Amersham Pharmacia Biotech, Uppsala, Sweden). Relative band densities were determined using an Image J computerized densitometry system. The experiments were carried out in triplicate measurements.

### 4.8. RNA Extraction and RT-PCR

RNA from A549 cells was extracted using the TRIZOL reagent (Invitrogen Corp., Carlsbad, CA, USA) according to the manufacturer guidelines. 1 μg of RNA was synthesized into cDNA with Maxime RT premix (Invitrogen Corp.) at 45 °C for 60 min and then at 95 °C for 5 min. Then, analysis of gene expression was performed in triplicates using a Maxime PCR premix kit (Invitrogen Corp.). Electrophoresed PCR products were visualized under UV light after ethidium bromide staining. The relative expression levels of target genes were normalized using GAPDH as an internal control. Relative band densities were determined using an Image J computerized densitometry system.

### 4.9. Statistical Analysis

Significance was determined by one-way analysis of variance (ANOVA) and Tukey’s multiple comparison tests. In all analyses, *p* < 0.05 was taken to indicate statistical significance.

## Figures and Tables

**Table 1:** Biological processes related to targets of MEO. Modules were assigned to biological processes using KEGG pathway 2019 human.

Module	KEGG Pathway 2019 Human	*p* Value	GO Biological Process 2018	*p* Value
M1	Mineral absorption	6.5 × 10^−4^	calcium ion transport (GO:0006816)	2.5 × 10^−22^
M2	Cytokine–cytokine receptor interaction	2.8 × 10^−16^	cellular response to cytokine stimulus (GO:0071345)	1.5 × 10^−^^14^
M3	Retinol metabolism	1.1 × 10^−4^	coumarin metabolic process (GO:0009804)	1.5 × 10^−3^
M4	Other glycan degradation	3.6 × 10^−3^	positive regulation of action potential (GO:0045760)	1.2 × 10^−3^
M5	Legionellosis	1.1 × 10^−6^	response to organic cyclic compound (GO:0014070)	3.2 × 10^−4^
M6	Th1 and Th2 cell differentiation	4.3 × 10^−4^	positive regulation of T-helper 17 type immune response (GO:2000318)	2.1 × 10^−3^
M7	Neuroactive ligand-receptor interaction	9.4 × 10^−5^	positive regulation of cytosolic calcium ion concentration (GO:0007204)	2.4 × 10^−6^

**Table 2:** Cluster of the enriched pathway of MEO.

Cluster No.	KEGG Pathway 2019 Human	*p* Value
Cluster 1	Cytokine–cytokine receptor interaction	2.779 × 10^−16^
Cluster 2	JAK-STAT signaling pathway	6.906 × 10^−^^16^
Cluster 3	Inflammatory bowel disease (IBD)	1.932 × 10^−^^13^
Cluster 4	Intestinal immune network for IgA production	1.604 × 10^−^^11^
Cluster 5	Autoimmune thyroid disease	2.685 × 10^−^^11^
Cluster 6	IL-17 signaling pathway	4.823 × 10^−^^10^
Cluster 7	Hematopoietic cell lineage	5.976 × 10^−^^10^
Cluster 8	T cell receptor signaling pathway	7.339 × 10^−^^10^
Cluster 9	Asthma	9.848 × 10^−^^10^
Cluster 10	Allograft rejection	2.307 × 10^−^^9^

## Data Availability

The data presented in this study are available on request from the corresponding author.

## Supplementary Materials

The following are available online at https://www.mdpi.com/1424-8247/14/1/2/s1. Table S1: Total target genes of menthol and menthone, major compounds of *Mentha piperita*., Table S2: Detailed information of Modules with Node IDs.
