# Supplementary material for: Inhalation of Essential Oil from Mentha piperita Ameliorates PM10-Exposed Asthma by Targeting IL-6/JAK2/STAT3 Pathway Based on a Network Pharmacological Analysis
Source: Pharmaceuticals (Basel). 2020 Dec 22;14(1):2. doi: 10.3390/ph14010002 (PMC7821947; doi:10.3390/ph14010002)
Supplement: Supplementary file 1 [file pharmaceuticals-14-00002-s001.zip › Supplementary Table1.docx]

Table S1. Total target genes of menthol and menthone, major compounds of Mentha *Mentha piperita*.

| **Compound Name** | **Co-Efficient Genes** |
| --- | --- |
| (-)-menthol | (+)-Menthofuran Synthase |
| (-)-menthol | (+)-Neomenthol Dehydrogenase |
| (-)-menthol | (+)-Neomenthol Dehydrogenase |
| (-)-menthol | 1-Aminocyclopropane-1-Carboxylate Oxidase Homolog 5 |
| (-)-menthol | 2-Alkenal Reductase (Nadp(+)-Dependent) |
| (-)-menthol | 3-Ketoacyl-Coa Synthase 6 |
| (-)-menthol | Cytochrome P450 2c3 |
| (-)-menthol | Ent-Kaurene Oxidase, Chloroplastic |
| (-)-menthol | Fos Proto-Oncogene, Ap-1 Transcription Factor Subunit |
| (-)-menthol | Indole-3-Glycerol Phosphate Synthase |
| (-)-menthol | Lipase E, Hormone Sensitive Type |
| (-)-menthol | Monoterpene Epsilon-Lactone Hydrolase |
| (-)-menthol | Mucin 5ac, Oligomeric Mucus/Gel-Forming |
| (-)-menthol | Myoblast City |
| (-)-menthol | Olfactory Receptor Family 2 Subfamily Ag Member 1 |
| (-)-menthol | Phenylalanine Ammonia-Lyase |
| (-)-menthol | Phosphoinositide Interacting Regulator Of Transient Receptor Potential Channels |
| (-)-menthol | Potassium Calcium-Activated Channel Subfamily M Alpha 1 |
| (-)-menthol | Potassium Two Pore Domain Channel Subfamily K Member 2 |
| (-)-menthol | Potassium Two Pore Domain Channel Subfamily K Member 4 |
| (-)-menthol | Probable Serine/Threonine-Protein Kinase Fhkc |
| (-)-menthol | Protein Eceriferum 2 |
| (-)-menthol | Protein Kinase X-Linked |
| (-)-menthol | Protein Narrow Leaf 1 |
| (-)-menthol | Ras-Related Protein Rapa |
| (-)-menthol | Short-Chain Dehydrogenase Srde |
| (-)-menthol | Sodium Channel Epithelial 1 Subunit Gamma |
| (-)-menthol | Transient Receptor Potential Cation Channel Subfamily A Member 1 |
| (-)-menthol | Transient Receptor Potential Cation Channel Subfamily C Member 1 |
| (-)-menthol | Transient Receptor Potential Cation Channel Subfamily C Member 5 |
| (-)-menthol | Transient Receptor Potential Cation Channel Subfamily C Member 6 |
| (-)-menthol | Transient Receptor Potential Cation Channel Subfamily V Member 2 |
| (-)-menthol | Transient Receptor Potential Cation Channel Trpm |
| (-)-menthol | Trk System Potassium Uptake Protein Trka |
| (-)-menthol | Unspecific Monooxygenase |
| (-)-menthol | Xenobiotic-Transporting Atpase |
| (-)-menthol | Acetylcholinesterase |
| (-)-menthol | Activating Transcription Factor 3 |
| (-)-menthol | Ankyrin 1 |
| (-)-menthol | Atpase Plasma Membrane Ca2+ Transporting 1 |
| (-)-menthol | C-X-C Motif Chemokine Ligand 1 |
| (-)-menthol | C-X-C Motif Chemokine Ligand 8 |
| (-)-menthol | Cadherin 1 |
| (-)-menthol | Calcitonin Related Polypeptide Alpha |
| (-)-menthol | Calcium Voltage-Gated Channel Subunit Alpha1 B |
| (-)-menthol | Calcium Voltage-Gated Channel Subunit Alpha1 I |
| (-)-menthol | Calumenin |
| (-)-menthol | Camp-Dependent Protein Kinase |
| (-)-menthol | Carbonic Anhydrase 1 |
| (-)-menthol | Catalase |
| (-)-menthol | Cation Channel Sperm Associated 3 |
| (-)-menthol | Claudin 1 |
| (-)-menthol | Cytochrome P450 Family 2 Subfamily A Member 6 |
| (-)-menthol | Dynactin Subunit 6 |
| (-)-menthol | G Protein-Coupled Receptor Class C Group 5 Member A |
| (-)-menthol | Glucose-6-Phosphate 1-Dehydrogenase |
| (-)-menthol | Glucosylceramidase Beta |
| (-)-menthol | Guanylate Cyclase |
| (-)-menthol | Hydroxymethylglutaryl-Coa Reductase |
| (-)-menthol | Hyperpolarization Activated Cyclic Nucleotide Gated Potassium And Sodium Channel 2 |
| (-)-menthol | Hyperpolarization Activated Cyclic Nucleotide Gated Potassium Channel 1 |
| (-)-menthol | Hypoxanthine Phosphoribosyltransferase 1 |
| (-)-menthol | Insulin |
| (-)-menthol | Interleukin 10 |
| (-)-menthol | Interleukin-6 |
| (-)-menthol | Kininogen 1 |
| (-)-menthol | Microseminoprotein, Prostate Associated |
| (-)-menthol | Mitogen-Activated Protein Kinase |
| (-)-menthol | Myeloperoxidase |
| (-)-menthol | Nerve Growth Factor |
| (-)-menthol | Neurturin |
| (-)-menthol | Phospholipase A(2) |
| (-)-menthol | Phospholipase A2 Group Vi |
| (-)-menthol | Phospholipase C |
| (-)-menthol | Potassium Voltage-Gated Channel Subfamily A Member 3 |
| (-)-menthol | Protein Kinase C Theta |
| (-)-menthol | Purinergic Receptor P2x 2 |
| (-)-menthol | Purinergic Receptor P2x 3 |
| (-)-menthol | Ribulose-5-Phosphate-3-Epimerase |
| (-)-menthol | Sodium Voltage-Gated Channel Alpha Subunit 10 |
| (-)-menthol | Solute Carrier Family 17 Member 8 |
| (-)-menthol | Tachykinin Precursor 1 |
| (-)-menthol | Taste 1 Receptor Member 3 |
| (-)-menthol | Taste 2 Receptor Member 38 |
| (-)-menthol | Transient Receptor Potential Cation Channel Subfamily M Member 1 |
| (-)-menthol | Transient Receptor Potential Cation Channel Subfamily M Member 3 |
| (-)-menthol | Transient Receptor Potential Cation Channel Subfamily M Member 4 |
| (-)-menthol | Transient Receptor Potential Cation Channel Subfamily M Member 5 |
| (-)-menthol | Transient Receptor Potential Cation Channel Subfamily M Member 6 |
| (-)-menthol | Transient Receptor Potential Cation Channel Subfamily M Member 7 |
| (-)-menthol | Transient Receptor Potential Cation Channel Subfamily M Member 8 |
| (-)-menthol | Transient Receptor Potential Cation Channel Subfamily V Member 1 |
| (-)-menthol | Transient Receptor Potential Cation Channel Subfamily V Member 3 |
| (-)-menthol | Transient Receptor Potential Cation Channel Subfamily V Member 4 |
| (-)-menthol | Transient Receptor Potential Cation Channel Subfamily V Member 5 |
| (-)-menthol | Transient Receptor Potential Cation Channel Subfamily V Member 6 |
| (-)-menthol | Tumor Necrosis Factor |
| (-)-menthol | Udp Glucuronosyltransferase Family 2 Member B7 |
| (-)-menthol | Udp-Glucuronosyltransferase |
| (-)-menthol | Uncoupling Protein 1 |
| menthone | (-)-Isopiperitenone Reductase |
| menthone | (+)-Menthofuran Synthase |
| menthone | (+)-Neomenthol Dehydrogenase |
| menthone | (+)-Neomenthol Dehydrogenase |
| menthone | (2e,6e)-Farnesyl Diphosphate Synthase |
| menthone | 1-Deoxy-D-Xylulose-5-Phosphate Synthase |
| menthone | 2-Alkenal Reductase (Nadp(+)-Dependent) |
| menthone | Adenosinetriphosphatase |
| menthone | Alpha,Alpha-Trehalose-Phosphate Synthase (Udp-Forming) |
| menthone | Alpha2u Globulin |
| menthone | Atp Binding Cassette Subfamily G Member 2 (Junior Blood Group) |
| menthone | Btb/Poz And Taz Domain-Containing Protein 2 |
| menthone | Btb/Poz And Taz Domain-Containing Protein 3 |
| menthone | Calcium-Transporting Atpase, Endoplasmic Reticulum-Type |
| menthone | Chorismate Mutase 1, Chloroplastic |
| menthone | Corticostatin-3 |
| menthone | Cytoglobin-1 |
| menthone | Dihydroflavanol 4-Reductase |
| menthone | Dimethylallyltranstransferase |
| menthone | Monocyclic Monoterpene Ketone Monooxygenase |
| menthone | Monoterpene Epsilon-Lactone Hydrolase |
| menthone | Myoblast City |
| menthone | Myopia 3 (High Grade, Autosomal Dominant) |
| menthone | Nadph--Hemoprotein Reductase |
| menthone | Phenylacetone Monooxygenase |
| menthone | Phenylalanine Ammonia-Lyase |
| menthone | Protein Arom |
| menthone | Protein Red1 |
| menthone | Ribulose-Bisphosphate Carboxylase |
| menthone | Tropinone Reductase Homolog At2g29330 |
| menthone | Unspecific Monooxygenase |
| menthone | Urease |
| menthone | Xenobiotic-Transporting Atpase |
| menthone | Acetylcholinesterase |
| menthone | Activin A Receptor Like Type 1 |
| menthone | Adenylate Cyclase |
| menthone | Alkaline Phosphatase |
| menthone | Alpha-Amylase |
| menthone | Atpase Plasma Membrane Ca2+ Transporting 1 |
| menthone | Beta-N-Acetylhexosaminidase |
| menthone | Beta-Trcp |
| menthone | Breast Cancer 3 |
| menthone | Butyrylcholinesterase |
| menthone | C-C Motif Chemokine Ligand 4 |
| menthone | Caspase 1 |
| menthone | Catalase |
| menthone | Colony Stimulating Factor 1 |
| menthone | Colony Stimulating Factor 2 |
| menthone | Deleted In Azoospermia Like |
| menthone | Dna Nucleotidylexotransferase |
| menthone | Farnesyl Diphosphate Synthase |
| menthone | Fizzy And Cell Division Cycle 20 Related 1 |
| menthone | Glucose-6-Phosphatase |
| menthone | Guanine Nucleotide-Binding Protein Subunit Beta |
| menthone | Heparanase |
| menthone | Hydroxysteroid 17-Beta Dehydrogenase 6 |
| menthone | Insulin |
| menthone | Interferon Alpha 1 |
| menthone | Interferon Lambda Receptor 1 |
| menthone | Interleukin 1 Receptor Associated Kinase 4 |
| menthone | Interleukin 10 |
| menthone | Interleukin 13 |
| menthone | Interleukin 18 |
| menthone | Interleukin 2 |
| menthone | Interleukin 4 |
| menthone | Interleukin 5 |
| menthone | Interleukin-6 |
| menthone | Internal Protein I |
| menthone | Maltase-Glucoamylase |
| menthone | Methyl-Cpg Binding Domain Protein 2 |
| menthone | Mitogen-Activated Protein Kinase |
| menthone | Mitogen-Activated Protein Kinase 14 |
| menthone | Mitogen-Activated Protein Kinase 3 |
| menthone | Mitogen-Activated Protein Kinase 8 |
| menthone | Nlr Family Pyrin Domain Containing 3 |
| menthone | Pancreatic Lipase |
| menthone | Peroxidase |
| menthone | Protein Mel-28 |
| menthone | Pyrimidinergic Receptor P2y4 |
| menthone | Rhodopsin |
| menthone | Sarcoglycan Beta |
| menthone | Toll Like Receptor 3 |
| menthone | Transient Receptor Potential Cation Channel Subfamily M Member 8 |
| menthone | Trna N6-Adenosine Threonylcarbamoyltransferase |
| menthone | Tumor Necrosis Factor |
| menthone | Tyrosine Hydroxylase |
