# Supplementary material for: Inhalation of Essential Oil from Mentha piperita Ameliorates PM10-Exposed Asthma by Targeting IL-6/JAK2/STAT3 Pathway Based on a Network Pharmacological Analysis
Source: Pharmaceuticals (Basel). 2020 Dec 22;14(1):2. doi: 10.3390/ph14010002 (PMC7821947; doi:10.3390/ph14010002)
Supplement: Supplementary file 1 [file pharmaceuticals-14-00002-s001.zip › Supplementary Table2.docx]

Table S2. Detailed information of Modules with Node IDs

| Module | KEGG pathway 2019 Human | p value | GO Biological Process 2018 | p value | Node IDs |
| --- | --- | --- | --- | --- | --- |
| M1 | Mineral absorption | 0.0006553 | calcium ion transport (GO:0006816) | 2.50E-22 | HCN4, CATSPER2, CACNA1B, CACNA1I, CATSPER3, HCN1, HCN2, SCN10A, TRPM1, TRPM3, TRPM4, TRPM5, TRPM6, TRPM7, TRPM8 |
| M2 | Cytokine-cytokine receptor interaction | 2.80E-16 | cellular response to cytokine stimulus (GO:0071345) | 1.51E-14 | GUCA2A, CSF1, CSF2, IFNA1, IL10, IL13, IL2, IL4, IL5, IL6 |
| M3 | Retinol metabolism | 0.0001098 | coumarin metabolic process (GO:0009804) | 0.001499 | BCHE, CAT, CYP2A6, G6PC, HSD17B6 |
| M4 | Other glycan degradation | 0.003595 | positive regulation of action potential (GO:0045760) | 0.0012 | AMY2A, GBA, HEXDC, HPSE |
| M5 | Legionellosis | 1.091E-06 | response to organic cyclic compound (GO:0014070) | 0.0003241 | MSMB, LPO, ALPL, CASP1, CDH1, CXCL1, IL18, PNLIP |
| M6 | Th1 and Th2 cell differentiation | 0.000433 | positive regulation of T-helper 17 type immune response (GO:2000318) | 0.002098 | IL4R, PKIA, CALCB, ANK1, DAZL, PRKCQ, UGT2B7 |
| M7 | Neuroactive ligand-receptor interaction | 0.0000944 | positive regulation of cytosolic calcium ion concentration (GO:0007204) | 0.00000241 | MPDZ, TAC3, PLBD1, SGCZ, OSGEP, ATP2B1, CXCL8, G6PD, HPRT1, INS, LPAR2, MAPK14, P2RX2, P2RY4, TRPV4 |
